# A Validated Reverse Phase HPLC Analytical Method for Quantitation of Glycoalkaloids in *Solanum lycocarpum* and Its Extracts

**DOI:** 10.1155/2012/947836

**Published:** 2012-03-27

**Authors:** Renata Fabiane Jorge Tiossi, Mariza Abreu Miranda, João Paulo Barreto de Sousa, Fabíola Silva Garcia Praça, Maria Vitória Lopes Badra Bentley, James Dewey McChesney, Jairo Kenupp Bastos

**Affiliations:** ^1^Laboratório de Farmacognosia, Faculdade de Ciências Farmacêuticas de Ribeirão Preto, Universidade de São Paulo, Avenida do Café, s/n, 14040-903 Ribeirão Preto, SP, Brazil; ^2^Arbor Therapeutics, LLC, 145 County Road 245, Etta, MS 38627, USA

## Abstract

*Solanum lycocarpum* (Solanaceae) is native to the Brazilian Cerrado. Fruits of this species contain the glycoalkaloids solasonine (SN) and solamargine (SM), which display antiparasitic and anticancer properties. A method has been developed for the extraction and HPLC-UV analysis of the SN and SM in different parts of *S. lycocarpum*, mainly comprising ripe and unripe fruits, leaf, and stem. This analytical method was validated and gave good detection response with linearity over a dynamic range of 0.77–1000.00 **μ**g mL^−1^ and recovery in the range of 80.92–91.71%, allowing a reliable quantitation of the target compounds. Unripe fruits displayed higher concentrations of glycoalkaloids (1.04% ± 0.01 of SN and 0.69% ± 0.00 of SM) than the ripe fruits (0.83% ± 0.02 of SN and 0.60% ± 0.01 of SM). Quantitation of glycoalkaloids in the alkaloidic extract gave 45.09% ± 1.14 of SN and 44.37% ± 0.60 of SM, respectively.

## 1. Introduction

*Solanum lycocarpum *A. St.-Hil. (Solanaceae), popularly known as wolf-fruit, is native to the Brazilian Cerrado and is used in folk medicine as an anti-inflammatory, a hypocholesterolemic, a hypoglycemic, a sedative, a diuretic, an antispasmodic, an antiepileptic, and to control obesity [1]. It is also utilized as an edible fruit in rural areas [2].

This species belongs to the genus Solanum, the largest genus of the family Solanaceae. This genus is distinguished by its variety and productivity of alkaloids; those combined with sugar moieties are known as glycoalkaloids [3, 4]. Certain glycoalkaloids, such as solasonine (SN) and solamargine (SM), stand out economically because their chemical structures are very similar to steroidal hormones and therefore have been proposed to be used as an important source for the production of medicines, such as contraceptives and steroidal anti-inflammatory drugs [5]. However, these compounds might also act as possible antiandrogenic agents and if they cross the placental barrier they could exert a possible effect on development of the embryo [6].

**Figure 1:**
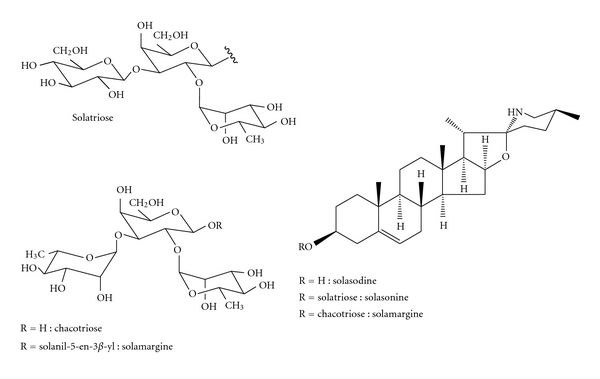
Chemical structures of solasodine and its respective glycoalkaloids.

From the alkaloidic extract of the fruits of *S. lycocarpum* was obtained a mixture of mostly the glycoalkaloids solasonine (SN) and solamargine (SM) (Figure 1). These compounds bear the same aglycone, solasodine, and differ from each other only in the nature of the involved trioses, namely, solatriose for solasonine and chacotriose for solamargine. These glycoalkaloids have been studied for their antidiabetic [7], antifungal [8], antiparasitic [9], and mostly for their anticancer properties [10, 11], including *in vivo* human nonmelanoma anticancer [12]. SN and SM showed selective cytotoxicity against cancer cells in relation to normal cells [13]. Therefore, considering the potential of *Solanum lycocarpum *as a medicinal plant, as a source of compounds for the synthesis of steroids and the development of new pharmaceuticals, as well as the lack of reliable quantitation methods for these compounds, the development of analytical methods for the quantitation of steroidal alkaloids in plant biomass, extracts, and final products would seem mandatory.

The aim of this paper was to develop a validated method to quantify the glycoalkaloids solasonine and solamargine in different plant tissues: leaves, stem, ripe and unripe fruits, as well as in the crude plant extracts.

## 2. Experimental Part

### 2.1. Chemicals and Reagents

HPLC grade acetonitrile (MeCN) and methanol (MeOH) were obtained from *Mallinckrodt Co.* (Xalostoc, Mexico). Anhydrous disodium hydrogen phosphate was acquired from *Carlo Erba Reagents* (Brazil), and deionized water was purified by *Milli-Q-plus* filter systems (Millipore, Bedford, MA, USA). Analytical grade ethanol (EtOH) and methanol were purchased from *Synth* (Brazil). Veratraldehyde (3,4-dimethoxybenzaldehyde), internal standard, was provided by *Merck* (Darmstadt, Germany). Solanine, a glycoalkaloid used as secondary standard, was bought from *Sigma-Aldrich Inc*. (St. Louis, MO, USA). Authentic compounds, solamargine and solasonine, were kindly provided by Dr. James D. McChesney from Ironstone Separations, Inc with purities estimated to be greater than 96% for both specimens.

### 2.2. Plant Material and Extracts Preparation

The leaves, stems, and ripe and unripe fruits of *S. lycocarpum *were collected in Cajurú, state of São Paulo, Brazil, in January of 2008. The plant material was authenticated by Professor Dr. Milton Groppo, Department of Botany, *Faculdade de Filosofia Ciências e Letras de Ribeirão Preto*, University of São Paulo, SP, Brazil, where a voucher specimen was deposited (SPFR: 11638). Chopped fruits, leaves, and branches were dried under air circulation in an oven at 45°C and powdered in a hammer mill. The particle size was standardized (mesh 35), and the powdered plant materials were stored in a sealed container in the freezer (−18°C) until use.

A neutral extract was obtained by maceration of 100 g of powdered dried fruit biomass with 200 mL of ethanol/water (80%) at room temperature for 72 h, three times, followed by percolation. The filtered extracts were combined and concentrated under vacuum furnishing 13.64 g of the crude hydroalcoholic extract (13.64% of extractable material).

The alkaloidic extract was prepared using a selective extraction based on the method of Henriques et al. [14]. The powdered dried fruits (1.0 kg) of *S. lycocarpum* were submitted to hydrochloric acid (0.2 M) extraction overnight by maceration, followed by filtration. Then, the aqueous acid extract was basified to pH 12.0 using 6.0 M NaOH. After precipitation the supernatant was removed; the precipitated material was centrifuged and the pellet was suspended in ethanol with shaking. The ethanol soluble fraction was concentrated under vacuum and lyophilized to furnish the alkaloidic extract (15.8 g).

### 2.3. Total Ash and Moisture Content

The total ash content of dried plant biomass was obtained by incinerating the samples using the methodology employed by Matos, [15]. The total moisture content was determined by loss on drying as described in AOAC, [16], and the data were collected in triplicate.

### 2.4. Analytical Method Conditions and Sample Preparation

A high performance liquid chromatography (HPLC), Shimadzu (Kyoto, Japan) instrument consisting of a UV detector, multisolvent delivery system (LC-10AD), autosampler (SIL-10ADvp), controller module (SCL-10Avp), autosampler and Class VP 5.02 software was used. A Zorbax SB-C18 analytical reverse phase column (250 × 4.6 mm i.d.; particule size 5 *μ*m) (*Agilent Technologies, USA),* coupled with a guard column from the same company was used.

The sample analyses were carried out employing an isocratic elution system using a mobile phase composed of acetonitrile and sodium phosphate buffer (pH 7.2; 0.01 M) in a ratio of 36.5 : 63.5 (v/v) at a flow rate of 1 mL/min. A 20 *μ*L aliquot of each sample was injected and a run time of analysis of 20 min with detection at 200 nm was employed.

For the quantitation of solasonine and solamargine in plant biomass, an aliquot of 250 mg of the powdered material were extracted in three replicates in a shaker (120 rpm/30°C/2 h), using 20 mL of 80% aqueous EtOH containing 3 *μ*g mL^−1^ of veratraldehyde (IS). For both the dried crude hydroalcoholic extract (42 mg) and the dried alkaloidic extract (4 mg), the samples were directly dissolved in 20 mL of IS solution.

All the samples were filtered and analyzed by HPLC-UV using the same conditions according to the analytical method developed. After analysis, the areas corresponding to veratraldehyde (IS), solamargine and solasonine were used to quantify the target compounds in the dried plant biomass. The results are reported as means ± S.D (standard error of the mean). The difference between the content in ripe and unripe fruits was determined using the unpaired *t*-test. Significant differences were considered for *P* values < 0.05.

### 2.5. Validation Parameters

**Figure 2:**
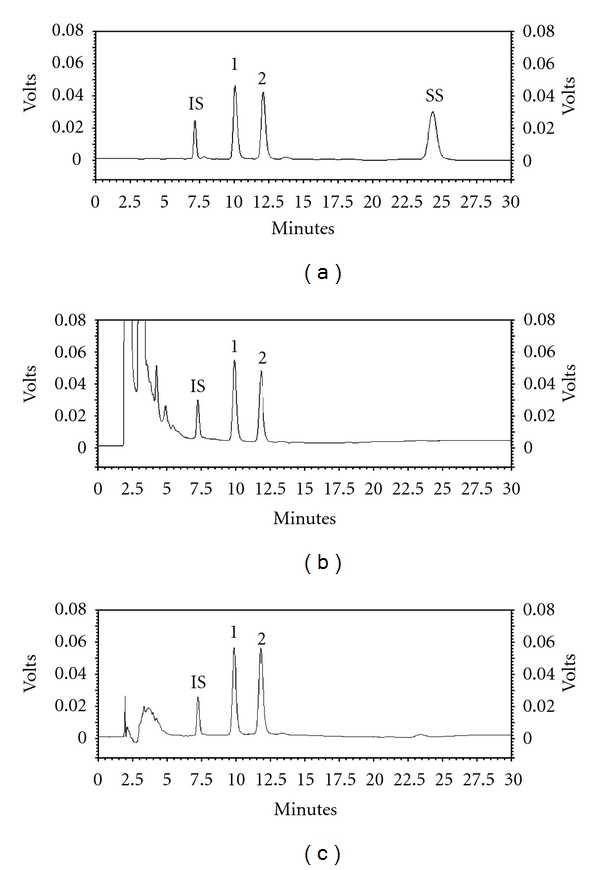
Chromatographic profiles by HPLC-UV: (a) standard compounds (b) hydroalcoholic extract of *Solanum lycocarpum* fruits (EtOH 80%) and (c) alkaloidic extract obtained from* Solanum lycocarpum* fruits. (IS) veratraldehyde, (1) solasonine, (2) solamargine, and (SS) solanine.

A validated analytical method was developed for the quantitation of the glycoalkaloids solasonine and solamargine in both plant biomass and extracts of *S. lycocarpum*, considering the parameters described by Ribani et al. [17], ANVISA [18], and ICH, [19]. Thus, selectivity was performed by comparing the chromatographic profiles of the analytical standards in relation to those obtained for plant biomass samples. For that purpose, the identification of the peaks was assured according to their retention times and by coelution with authentic standards. Veratraldehyde (rt = 7.2 min) was used as internal standard (IS), and it was added to the extracting solvent prior to the extraction (Figure 2).

The analytical curves of the standards were prepared in concentrations ranging between 0.77 and 1000.0 *μ*g mL^−1^ for both glycoalkaloids and between 1.62 and 12.5 *μ*g mL^−1^ for veratraldehyde. These solutions were analyzed in triplicate using the above analytical method. The correlation coefficients (*r*) were determined for each compound, and linear regression obtained and its correlations were used for the deduction of equations to quantify the glycoalkaloids.

The limits of detection (LOD) and quantification (LOQ) were determined based on the parameters of the analytical curves, considering standard deviation of the response (*s*) and the slope of the analytical curve (*S*). Thus, the curves were made in triplicate and values of *s* and *S* were applied at equations LD = 3.3 × *s*/*S* and LQ = 10 ×* s*/*S* [20].

The precisions were determined by the evaluation of the repeatability (intraday) and by intermediate precision (interday). Repeatability was determined by preparation and analysis of the same sample in six replicates evaluated on the same day and by the same technician. Intermediate precision was also performed in six replicates at intervals of one day with injections made by two different technicians. The data obtained were expressed as the relative standard deviation (RSD %).

Accuracy was evaluated by recovery studies using a method of spiking with the chemical markers a previously exhausted matrix consisting of dried and powdered *S. lycocarpum* leaves (250 mg). For that, 35 g of plant biomass were exhaustively extracted with 96% aqueous ethanol using a Soxhlet apparatus to reduce the content of glycoalkaloids to trace amounts which was confirmed by HPLC analysis. After that, the matrix was spiked by adding the glycoalkaloids in solution, in four replicates, at three levels of concentration: low, medium, and high, corresponding to 62.5, 125.0 and 187.5 *μ*g mL^−1^, respectively. Then, the spiked matrix was extracted using 20 mL of extraction solvent consisting of 80% EtOH containing 3 *μ*g mL^−1^ of veratraldehyde. After extraction, 1 mL of secondary standard, solanine, at 300 *μ*g mL^−1^ was added to aliquots of 5 mL of each extract. The resultant solutions were filtered and analyzed by HPLC-UV, using the analytical method developed.

Robustness was determined using the test established in the literature [21]. For that, six factors including extraction time (A), sample size (B), particle size (C) extraction temperature (D), stirring (E), and extraction volume (F) were evaluated using a combination of eight factors to determine the possible variation in the sample preparation by using different conditions. In Table 1 the nominal factors are codified by capital letters and the variations are codified by lower-case letters. As displayed in Table 1, the combination of **1 **furnished the result *s*,and the consecutive results were obtained by taking into consideration the combinations **2**–**8**. To determine the variation of a factor, four values corresponding to capital letters and four values corresponding to lower-case letters were considered by statistical analysis comparing the average between the two groups. For example, for the influence of the factor A (extraction time), was evaluated by comparison of the mean group with capital letters [(*s *+ *t *+ *u *+ *v*)/4] and the mean group of lower-case [(*w *+ *x *+ *y *+ *z*)/4]. The difference between groups was determined by using the unpaired *t*-test. Significant differences were considered for *P* values < 0.01.

## 3. Results and Discussion

 Phytochemical studies have shown that *Solanum lycocarpum* fruit contain different classes of compounds, such as: phenols and tannins [22]; glycoalkaloids as solamargine, solasonine, 12-hydroxysolasonine, robeneoside A, and robeneoside B [7], as well as lobofrutoside and saponins, as lyconoside Ia, Ib, II, III, and IV [23]. However, this method has been developed for the glycoalkaloids solasonine and solamargine, not only because they are major compounds in *Solanum lycocarpum* fruits, but also because of their potential importance for the development of new pharmaceuticals.

 The determination of moisture and total ash content in plant biomass is of paramount importance for the quality control of medicinal plants, since high values of water favor the action of enzymes which can degrade the active ingredients, and enable the growth of microorganisms. Total ash determination allows the verification of nonvolatile inorganic impurities, which may include contaminants such as sand coming from a careless handling during the processing of plant material [24]. The maximum moisture content tolerated should be between 8 and 14% [25]. The contents of total ash and total moisture in the dried fruits of *S. lycocarpum* amounted to 2.52% ± 0.001 and 4.72% ± 0.774, respectively. Thus, considering the recommended values, the moisture content of our samples was acceptable.

 To ensure the quality of plant materials and their products it is necessary to apply validated analytical methods and ensure that the developed methods are selective, accurate, reproducible, and robust for the purposes they are designed [26].

 Evaluation for selectivity for solamargine and solasonine in the chromatograms of both standard compounds and hydroalcoholic extract revealed no peak of interference. The peaks were identified by comparing the retention times of the analyzed compounds with authentic standards. The retention times obtained for solasonine and solamargine were 10.08 min and 12.08 min, respectively (Figure 2). Also, the linear regression coefficients (Table 2) displayed values higher than 0.999 for all standard compounds. Both glycoalkaloids presented a wide linear dynamic range of 0.77–990.0 *μ*g mL^−1^ for SN and 0.78–1000.0 *μ*g mL^−1^ for SM. Quantification of the alkaloids was carried out using the linear regression equations obtained for both SN and SM, and linear regression of internal standard (Table 2).

The limits of detection and quantification obtained were 0.29 and 0.86 *μ*g mL^−1^ for solasonine, and 0.57 and 1.74 *μ*g mL^−1^ for solamargine, respectively, rendering the method sufficiently sensitive for the present purposes.

The values obtained for repeatability and intermediate precision (in RSD %) for precision ranged from 1.13 to 5.57% for concentrations, and between 2.16 and 2.55% for retention times of SN and SM, respectively (Table 3). Therefore, the developed method presents good precision [17, 18].

Recovery studies are very important to determine the accuracy of an analytical method. It ensures the quantification of target compounds measured [17]. In this regard, the obtained recoveries for solasonine, solamargine, and veratraldehyde (Table 4) were higher than 80.92%, reaching 91.71% of recovery with range RSD (%) of 0.77 to 5.14%, and error ranging between 8.29 and 19.08%, which indicates that the developed method displays good accuracy.

The robustness measures the sensitivity of the method to small experimental variations [21]. Thus, to assess the robustness of an analytical method it is necessary to evaluate the influence of small deliberate variations of the analytical method parameters on the result obtained. Then, recognizing that sample preparation and manipulation by the technician is an important source of error, that parameter was considered in the variables described in Table 1.

The influence of variability of each parameter was assessed by comparing the results obtained for the nominal conditions and for variations of these conditions. Then, these groups were compared regarding quantification of solasonine and solamargine in results obtained with deliberate variations of the parameters. No statistical difference between the studied groups was observed. Therefore, these data suggest that the developed method is reliable for the quantification of SN and SM considering the evaluated parameters.

No detectable amounts of solasonine and solamargine were found in stems and leaves of the species studied SL. These compounds were found in leaves of other *Solanum* species, such as: *S. xanthocarpum* [27], *S. havanense*, *S. scabrum*, *S. lycopersicoides* [3], and *S. sodomaeum *[28]. However, the fruits of SL bear a significant amount of both glycoalkaloids. Also, the amounts of glycoalkaloids were statistically different between ripe and unripe fruits. The unripe fruits displayed significantly higher concentrations of glycoalkaloids in comparison with ripe ones, which furnished, respectively, 1.04% and 0.83% of solasonine and 0.69% and 0.60% of solamargine (Table 5). Therefore, from the phytochemical point of view, these quantitative data suggest that when the objective is optimum retrieval attainment of glycoalkaloids, it is more profitable to harvest the fruit while it is still unripe.

Besides, solasonine and solamargine are present in more than 100 species of the genus *Solanum*, as for instance: *S. melongena* [4] and *S. incanum* [11]. However, it should be pointed out that the amounts of these alkaloids found in other *Solanum* species are not comparable with the amounts found in *S. lycocarpum*. For instance, the contents of solasonine and solamargine in mg per 100 g^−1^ in other* Solanum* species fruits were, respectively,* S. melongena* (0.17–1 and 0.58–4.5); *S. macrocarpum* (16–23 and 124–197); *S. aethiopicum*, on wet basis (0.41–1 and 0.58–4.86) [29]; *S. ptycanthum*, on wet basis (490 and 330) [30]; *S. sodomaeum*, as total glycoalkaloids (830 for ripe fruits and 450 for unripe fruits) [28].

 Although the fruits of *S. lycocarpum* display comparable nutritional values for their contents of sugars, vitamin C, and iron, with other edible fruits, such as banana, pineapple, and orange [2], it should be taken into consideration that the safety limits for the intake of glycoalkaloids from potato are 0.02% of fresh samples (estimate of 0.1% dried potato) [31]. Therefore, the amount of total glycoalkaloids in dried fruits of *S. lycocarpum*, 1.73% and 1.43%, for unripe and ripe fruits, respectively, indicates that the ingestion of these fruits could be toxic. Hence, the population should be advised of the risks associated with the intake of this fruit, especially for pregnant women, since there is reported fetotoxic effect in rats [31], which may affect fetus development [6]. Nevertheless, so far, no clinical evidence of maternal toxicity has been reported [32]. Moreover, *S. lycocarpum* has economical relevance not only for the synthesis of steroid derivatives, due to the presence of the steroidal moiety in these compounds, but also as raw material for the development of new pharmaceuticals.

Regarding the productivity and economic utilization of this species, we pointed out that SL plant can bear from 40 to 100 fruits per adult individual, and the weight of each fruit varies from 400 to 900 g [2]. Also, it bears fruits throughout the year, with the highest productivity between January and July [2, 33]. This crop does not require large investments, since it is able to grow and thrive in unfavorable environmental conditions, including low nutrients and acidic soils, as well as its ability to withstand a harsh climate and periods of prolonged drought, as a plant characteristic of the Brazilian Cerrado [34]. In addition, the fruit harvesting can be ecologically sustainable, since collecting fruits would not impact the environment, if well handled.

Phytochemical studies revealed that *S. lycocarpum *fruits, in addition to the glycoalkaloids, contain tannins and phenolics [22]. Also, sensorial studies showed that the bitterness or burning associated with the intake of *Solanum *fruits are related to glycoalkaloid content and not with the phenolic compounds [35]. High contents of glycoalkaloids would likely be noticed by humans making consumption of the fruits unacceptable [36]. Besides, other studies have reported that higher amount of glycoalkaloids in *Solanum* makes the fruit more toxic and less palatable to frugivorous seed dispersers [37]. Therefore, from the ecological point of view, the ripe fruit should be favored by both frugivorous which feed on less toxic fruits and the plant species by having its viable mature seeds spread.

The extraction protocol for alkaloids used in our work was very selective, because the content of glycoalkaloids in the obtained extract was quite impressive, corresponding approximately to 90% of the alkaloidic extract, while in both fruit dry biomass and its hydroalcoholic extract it corresponded to 1% and 10% of glycoalkaloids, respectively (Table 5).

In conclusion, we have developed a validated analytical method which is reliable and brings an important contribution to the field, since *S. lycocarpum* fruits do not only contain high amounts of glycoalkaloids and *S. lycocarpum* has a very good productivity of fruits which could also be used as a crop for the production of steroidal drugs and new pharmaceuticals.

## Figures and Tables

**Table 1:** Combination of factors to assess the robustness of method.

Factors	Nominal			Variation				
Extraction time	A, 2.0 h			a, 1.5 h				
Sample size	B, 250 mg			b, 225 mg				
Particle size	C, 35 mesh			c, 40 mesh				
Extraction temperature	D, 30°C			d, 40°C				
Stirring	E, 120 rpm			e, 150 rpm				
Extraction volume	F, 20.0 mL			f, 20.5 mL				

	Factors level							
Combination	**1**	**2**	**3**	**4**	**5**	**6**	**7**	**8**

*Experiments*								
A or a	A	A	A	A	a	a	a	a
B or b	B	B	b	b	B	B	b	b
C or c	C	c	C	c	C	c	C	c
D or d	D	D	d	d	d	d	D	D
E or e	E	e	E	e	e	E	e	E
F or f	F	f	f	F	F	f	f	F
Results	*s*	*t*	*u*	*v*	*w*	*x*	*y*	*z*

(*s*) Nominal conditions of method. Variables: (*t*) particle size, stirring, and extraction volume; (*u*) sample size, extraction temperature, and extraction volume; (*v*) sample size, particle size, extraction temperature, and stirring; (*w*) extraction time, extraction temperature, and stirring; (*x*) extraction time, particle size, extraction temperature, and extraction volume; (*y*) extraction time, sample size, stirring, and extraction volume; (*z*) extraction time, sample size, and particle size.

**Table 2:** Linear regression to quantify glycoalkaloids.

Compounds	Linear range	Linear coefficient (*a*)	Angular coefficient (*b*)	Correlation coefficient (*r*)
SN	0.77–990.00	5603.2	9026.0	0.9996
SM	0.78–1000.00	6433.3	9204.0	0.9996
IS	1.62–12.50	97525.0	1227.3	0.9998
SS	24.75–990.00	7996.1	8856.7	0.9992

Analysis with a regression equation of *y = ax + b*, in which *x* is the concentration in *μ*g mL^−1^,* y* is the peak area, *a* is angular coefficient, and *b* is linear coefficient. SN: solasonine, SM: solamargine, IS: internal standard (veratraldehyde), and SS: secondary standard (solanine).

**Table 3:** Repeatability and intermediate precision.

	Conc ± SD (*μ*g mL^−1^)	RSD (%)	RT ± SD	RSD (%)
*Repeatability*				
Solasonine	194.98 ± 6.01	3.08	10.3 ± 0.223	2.16
Solamargine	15.30 ± 1.77	1.13	12.3 ± 0.281	2.27
*Intermediate precision*				
Solasonine	189.38 ± 10.54	5.57	10.2 ± 0.255	2.48
Solamargine	157.04 ± 6.74	4.29	12.2 ± 0.313	2.55

Conc: concentration; SD: standard deviation; RSD: relative standard deviation; RT: retention time.

**Table 4:** Accuracy and recovery of solasonine, solamargine, and veratraldehyde.

	Conc (*μ*g mL^−1^)	MR (%) ± SD	RSD (%)	Error (%)
Solasonine				
Low	62.5	81.92 ± 0.63	0.77	18.08
Medium	125.0	90.01 ± 3.97	4.41	9.99
High	187.5	85.41 ± 4.39	5.14	14.59
Solamargine				
Low	62.5	80.92 ± 0.90	1.11	19.08
Medium	125.0	91.71 ± 4.51	4.92	8.29
High	187.5	88.31 ± 4.20	4.75	11.69
Veratraldehyde (IS)	3.0	84.12 ± 2.98	3.54	15.88

MR: mean recovery.

**Table 5:** Quantification of glycoalkaloids in different tissues of *Solanum lycocarpum *and in fruit extracts.

	Quantification (% ± SD)	
Sample	Solasonine	Solamargine
Leaves	nd	nd
Branch	nd	nd
Unripe fruit	1.04 ± 0.01*	0.69 ± 0.02*
Ripe fruit	0.83 ± 0.02*	0.60 ± 0.01*
Alkaloidic extract	45.09 ± 1.14	44.37 ± 0.60
Hydroalcoholic extract	6.63 ± 0.39	4.65 ± 0.40

nd: not detected, considering the LOD of this analytical method. *P < 0.05.
